# Parents' Perceptions of Claims on Packaged Commercial Toddler Foods: A Qualitative Study

**DOI:** 10.1111/mcn.70032

**Published:** 2025-04-22

**Authors:** Mamaru Ayenew Awoke, Maree Scully, Kerryn Alexander, Jennifer McCann, Alison McAleese, Anthea Rhodes, Jane Martin, Andrea Schmidtke, Helen Dixon

**Affiliations:** ^1^ Centre for Behavioural Research in Cancer, Cancer Council Victoria Melbourne Victoria Australia; ^2^ Kerryn Alexander Research Pty Ltd Melbourne Victoria Australia; ^3^ Institute for Physical Activity and Nutrition (IPAN), School of Exercise and Nutrition Sciences Deakin University Burwood Victoria Australia; ^4^ Prevention Division Cancer Council Victoria Melbourne Victoria Australia; ^5^ The Royal Children's Hospital Melbourne Parkville Victoria Australia; ^6^ Department of Paediatrics The University of Melbourne Parkville Victoria Australia; ^7^ Food for Health Alliance Melbourne Victoria Australia; ^8^ School of Psychological Sciences The University of Melbourne Parkville Victoria Australia

**Keywords:** child nutrition, claims, commercial toddler foods, food industry, parents, policy

## Abstract

Commercial toddler foods (CTFs) are widely used. Many are nutritionally poor despite displaying health, nutrition or marketing claims implying healthiness. This study aimed to: (i) explore how claims on CTFs influence parents' product perceptions and preferences, and gauge support for regulatory reform to ensure CTFs are marketed responsibly; (ii) identify other factors influencing parent's and toddlers' perceptions and preferences towards CTFs and toddler feeding in general.

Six moderator‐led online focus groups (*N* = 47) were conducted with parents of toddlers (aged 12 to < 36 months) in Victoria, Australia, using a discussion guide and mock CTFs to elicit discourse. Discussions were audio‐recorded, transcribed, and analysed using exploratory content and thematic analysis.

Parents found CTFs displaying claims more appealing, despite awareness and scepticism of claims. Parents expressed frustration over industry's use of claims and supported stronger regulation of claims. Some doubted the effectiveness of regulations, concerned that industry would find ways to circumvent rules. Parents perceive visual features (especially cartoon characters) on CTFs as highly influential for toddlers. Parents support honest labelling of CTFs, reflecting a product's actual ingredients and nutritional profile, especially signposting of sugar content. Parents evaluate CTFs based on perceived healthiness and convenience, often relying on social media and brand reputation for guidance on what to feed their toddlers.

While parents are aware of, and sceptical about claims on CTFs, these claims still influence their perceptions. Findings highlight the need for stronger regulations to ensure that claims on CTFs do not create inaccurate perceptions of their healthiness.

## Summary

- Parents find claims on CTFs appealing and influential, notwithstanding awareness and scepticism of such claims.
- ‘Free from “bad” ingredient’ claims (e.g., preservative‐free), which appeal to risk avoidance motivation, hold slightly greater appeal and influence on parents' product perceptions compared to other types of claims commonly featured on CTFs.
- Parents stressed the need for transparent and honest labelling of CTFs with regard to claims, as well as product names, ingredients lists and nutritional information.
- Many parents expressed strong concern about the sugar content in CTFs, supporting stricter limits and clearer signposting of sugar in CTFs to highlight potential harm.
- Many parents expressed strong support for stricter regulation of claims on CTFs to ensure that these products are marketed suitably and do not create inaccurate perceptions of product healthiness.

## Graphical abstract

Commercial toddler foods (CTFs) are widely used. Many are nutritionally poor despite displaying health, nutrition or marketing claims implying healthiness. This study used focus groups with parents of toddlers in Victoria, Australia, to explore how claims on CTFs influence parents' product perceptions and preferences, and gauge support for regulatory reform to ensure CTFs are marketed responsibly; and identify other factors influencing parent's and toddler's perceptions and preferences towards CTFs and toddler feeding in general. While parents are aware of, and sceptical about claims on CTFs, these claims still influence their perceptions, and the findings highlight the need for stronger regulations to ensure that claims on CTFs do not create inaccurate perceptions of their healthiness.

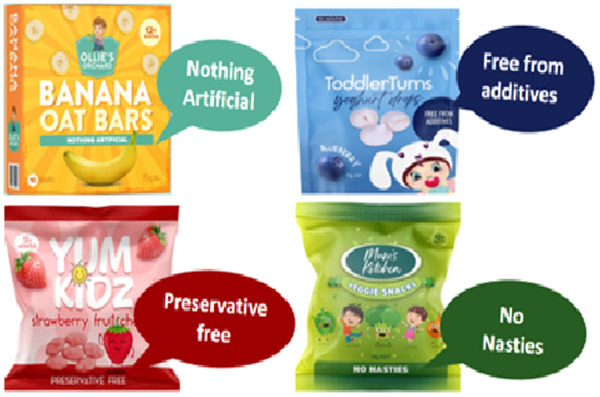

## 1 Introduction

Nutrition during early childhood (< 3 years) is essential for optimal growth and development. The Australian Dietary Guidelines recommend that toddlers (12–36 months) should consume a variety of nutritious foods from the five food groups (vegetables and legumes; grain (cereal); fruit; lean meats, nuts and; dairy and alternatives) limiting foods high in saturated fat, sugar, and salt (National Health and Medical Research Council 2003). In the Australian retail market, there has been a significant rise in the number of commercial toddler food (CTF) products, particularly snacks (McCann et al. 2021a; McCann et al. 2021b), and many parents rely on these products to feed their toddlers (The Royal Children's Hospital National Child Health Poll 2022). Concerningly, many CTFs are nutrient poor and high in sugar, and/or salt (Scully et al. 2023). A multi‐national audit of commercial baby and toddler foods found almost half were ultra‐processed, containing significantly higher sugar than less processed products (Pries et al. 2024). Ultra‐processed foods are designed to be highly profitable, convenient and palatable, bearing minimal similarities to whole or homemade foods, which they are liable to displace (Monteiro et al. 2019). Regular consumption of energy‐dense, nutrient poor (i.e., ‘unhealthy foods’) in early childhood increases the risk of short and long‐term health problems, including weight gain, obesity, and non‐communicable diseases later in life (Chang et al. 2021; Chen et al. 2020; Hu 2013; Leffa et al. 2020; Neri et al. 2022). Furthermore, early exposure to unhealthy foods can shape children's food preferences and impact their dietary choices in later life (Ventura and Worobey 2013). Thus, the early years of life are crucial for establishing healthy eating habits, including food preferences, acceptance, and intake to support physiological, functional, and cognitive processes (Birch et al. 2007; Scaglioni et al. 2018).

The Australia New Zealand's Food Standard Code (FSC) mandates stringent criteria for health claims on all food products (including CTFs), sets parameters for making nutrition content claims about some properties in foods (e.g., to make a ‘low sugar’ claim a food must have less than 5 g/100 g of sugars) and permits nutrition content claims that simply state the presence or absence of certain ingredients (e.g., “free from artificial flavours”) (Food Standards Australia New Zealand 2013), however, there is no specific regulation relating to other types of claims that also commonly appear on toddler foods (e.g., “perfect for tiny hands”). The World Health Organization's (WHO) nutrient and promotion profile model (NPPM) (WHO Regional Office for Europe, 2022) is underpinned by the “belief that the health and development of infants and young children is critically important” and that as such “usual rules governing product composition, *labelling and promotion* should, therefore not apply [to those foods]” To ensure commercial infant and toddler foods have suitable and clear marketing and labelling, the WHO recommends that these foods *do not* display any claims (health, nutrition or marketing claims—hereafter referred to collectively as ‘claims’) on food packaging (WHO Regional Office for Europe, 2022). In Australia, such claims appear extensively on commercial infant and toddler foods (Dunford et al. 2024; Scully et al. 2024).

Previous research has found that claims appearing on CTFs often imply superior nutritional content and essentiality over home‐prepared foods, or that they are crucial for healthy growth and development, raising concerns that such claims may mislead caregivers about the healthiness of CTFs and adversely affect their food purchasing preferences for their toddlers (García et al. 2019; McCann et al. 2021a) (García et al. 2019; McCann et al. 2022). It is crucial to deepen our understanding of how common claims on CTFs influence parents and the food choices they make for their toddlers. This can be used to inform policy responses to ensure these products are labelled and marketed suitably.

Previous experimental research found that front‐of‐pack (FOP) nutrition content claims can influence parents' and school‐aged children's preferences towards unhealthy foods displaying these claims (Dixon et al. 2011). Another study looking at toddler foods and milks, found that nutrition content claims were especially influential, but that claims related to convenience, taste, natural ingredients and age appropriateness were also associated with an increase in parent's perceptions of the healthiness of toddler foods and milks, providing a ‘health halo’ for these products (McCann et al. 2022). Richter et al. (2022) (Richter et al. 2022) found that claims about brain development or immunity on toddler milks can mislead parents and increase the appeal of these products. Our quantitative online experiment testing the effect of single FOP claims on CTFs on parents' product preferences, perceptions and purchasing intentions found that ‘child‐related claims’ and ‘contains “good” ingredient claims’ were influential for certain parent subgroups (sole parents and those with three or more children respectively), while ‘free from “bad” ingredients’ claims increased all parents' intentions to purchase CTFs displaying these claims—especially parents with three or more children (Dixon et al. 2024). Together these experiments demonstrate that FOP claims can influence parents' food product perceptions, preferences and purchasing.

Previous qualitative studies have explored factors influencing parents' product perceptions and decisions to purchase commercial foods (Isaacs et al. 2022; Ravikumar et al. 2022) and sugary drinks (Fleming‐Milici et al. 2022; Hoare et al. 2014) for their young children, and parents' views on FOP nutrition labelling schemes (Maubach and Hoek 2010). However, there is a gap in the qualitative literature concerning parent's subjective experience and views on claims on CTFs. Therefore, the primary aims of this study were to use qualitative methods to explore how claims on CTFs influence parents' product perceptions and preferences, and gauge support for regulatory reform to ensure CTFs are marketed responsibly, through addressing the following research questions:

a. How do common claims on CTFs influence parents' product perceptions and preferences?
b. How do parents feel about these claims once they are informed about how influential certain claims can be?
c. Would parents support stricter regulation of claims on CTFs? And
d. What other factors influence parents' and toddlers’ perceptions and preferences regarding CTFs and toddler feeding?

To help contextualise this study, secondary aims were to explore which factors parents consider to be most important in influencing their own and their toddler's appraisals of and preferences towards CTFs, and where parents prefer to access nutritional information and guidance about what to feed their toddler.

## 2 Methods

### 2.1 Participants

Online focus group discussions were conducted with parents and carers (hereafter collectively referred to as parents) of toddlers aged 12 to < 36 months residing in Victoria, Australia who are responsible for most food purchases for their household. Parents were recruited by a specialist research agency following a convenience sampling approach. This method involved selecting individuals who were readily accessible from the agency's established online panel and who met the study's eligibility criteria. The panel itself was constructed from a diverse pool of individuals who had previously expressed interest in participating in research studies, ensuring a broad representation of the target population. Parents were excluded from participating if they had taken part in a focus group or in‐depth interview within the past 3 months, or if they and/or their close family and friends worked in advertising, marketing, market research, health promotion, or food industries. Parents were offered an incentive of AUD$100 in line with current market rates for online focus groups. A total of six focus groups were conducted (*N* = 47), with five groups comprising eight parents each, and one group consisting of seven parents. The groups were stratified based on parents ‘education level (low—less than tertiary degree)/high—tertiary degree or higher) and parental status (sole/co‐parent) to ensure equal distribution across these factors. Most groups comprised more mothers than fathers, with 1–3 fathers and 5–6 mothers per group, which was also the case in our previous study (72% women vs. 28% men) (Dixon et al. 2024).

### 2.2 Materials

All focus groups were conducted using a discussion guide, which was designed to elicit parents' experiences and views pertaining to the research questions. The discussion guide presented key topics, probing questions, and prompts to facilitate a structured group discussion, while allowing scope for open‐ended conversation (Supporting Information S1: File S1). To optimise flow, the order of questions and prompts opened with a general discussion about toddler's food preferences, parent's preferences for what to buy to feed their toddler, and which product attributes they consider to be important in influencing their own and their child's evaluation and choice of CTFs (secondary aims). Next, a series of questions and prompts examined parents understanding and perceptions of claims that commonly appear on CTFs, and their views on regulation of these claims (primary aims, RQs 1–3). Final questions explored parent's views on additional regulatory options for CTFs, such as introducing stricter rules about the ingredients, or levels of certain nutrients (e.g., sugar) permitted in CTFs, or requiring FOP warning labels highlighting the presence of harmful nutrients, concluding with a brief discussion about parents' preferred information sources for guidance on feeding their toddler (secondary aims).

**Figure 1:**
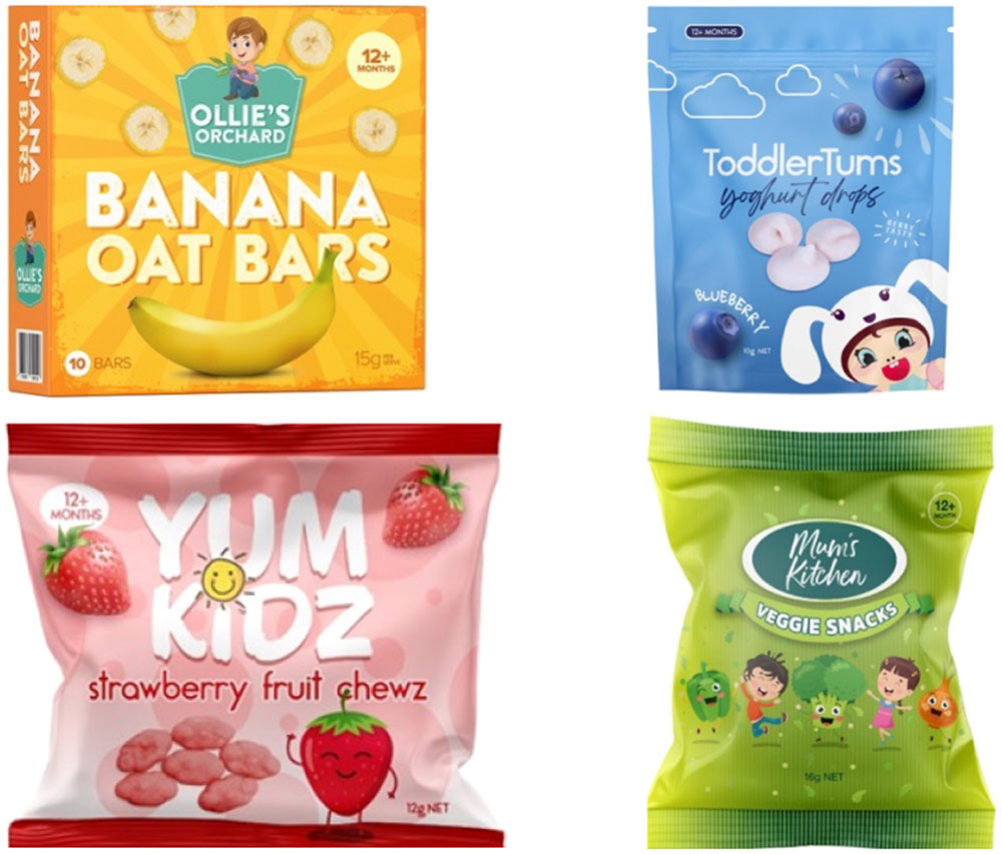
Toddler food packages without claims.

PowerPoint slides depicting full‐colour digital images of four mock CTFs displaying various common claims were used as stimuli. These mock products and claims were identical to those tested in our earlier quantitative experiment (Dixon et al. 2024). The product images were professionally prepared by a graphic designer to mimic top‐selling CTFs sold in Australia with key design features, such as brand names, fonts, and colours, altered to avoid participants’ responses being influenced by pre‐existing brand or product knowledge. Separate slides depicting each mock CTF were shown, with the presence of FOP claims varying as follows: (i) no claims present (control); (ii) ‘contains “good” ingredient’ claims (nutrition content claim focusing on benefit‐seeking motivational appeal, e.g., made with real fruit or whole grain); (iii) ‘free from “bad” ingredient’ claims (nutrition content claim focusing on risk‐avoidance motivational appeal, e.g., free from preservatives, no additives); (iv) child‐related claims (unregulated marketing claims highlighting developmental benefits appealing to parental motivations, e.g., encourages self‐feeding). See Figure 1 and Table 1.

**Table 1:** Illustrations of claim types tested in relation to CTFs and exemplary quotes from parents in response to these.

Claims tested across four CTF products	Quotes illustrating common responses by parents to CTFs displaying each claim type.
**‘Free from “bad” ingredients’ claims** 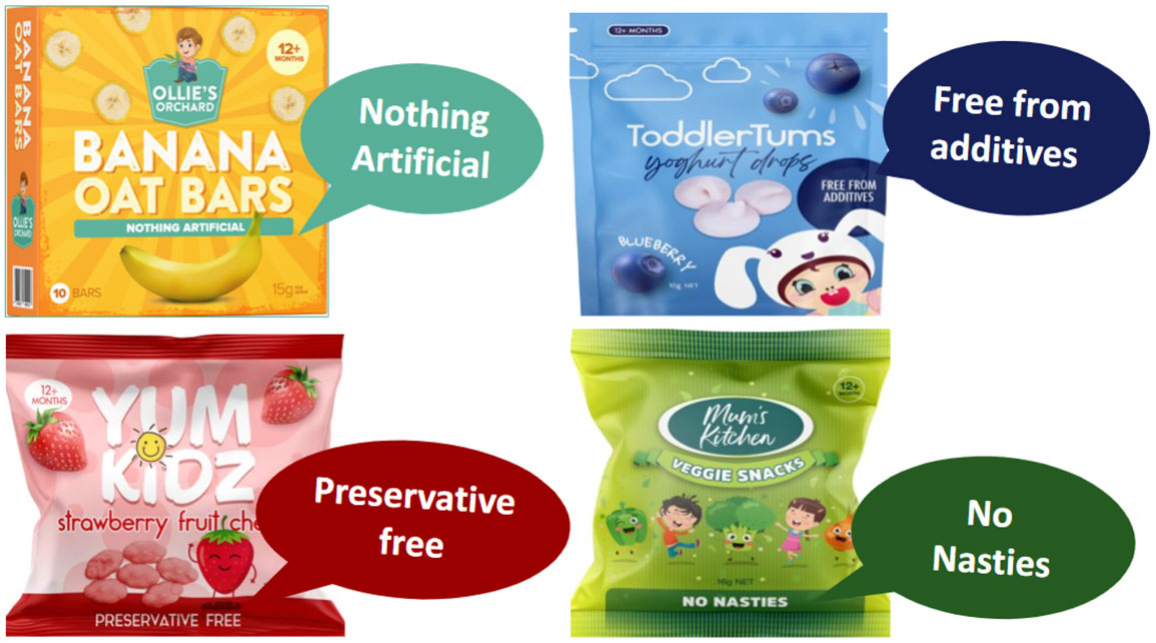	*“Yes, to me this is more productive marketing [compared to the other types of claims presented]. If something like this [‘free from “bad” ingredient’ claim] is on the label, I would be more likely to go and look at it rather than the previous line which said, ‘good for small hands’ or ‘easy chewable’ [child‐related claims].”*
	(G2, LE, co‐parent)
	*That [‘free from “bad” ingredient’ claim] seems like that is a much more concrete claim. If you are saying nothing artificial, well, it has to be true. The others seemed a little bit more misleading or a little bit more generalised whereas this seems a little bit more factual.*
	(G5, HE, coparent)
	*“I feel more reassured when I see things like that, especially the preservative free.”*
	(G6, HE, sole parent)
**‘Contains “good” ingredients’ claims** 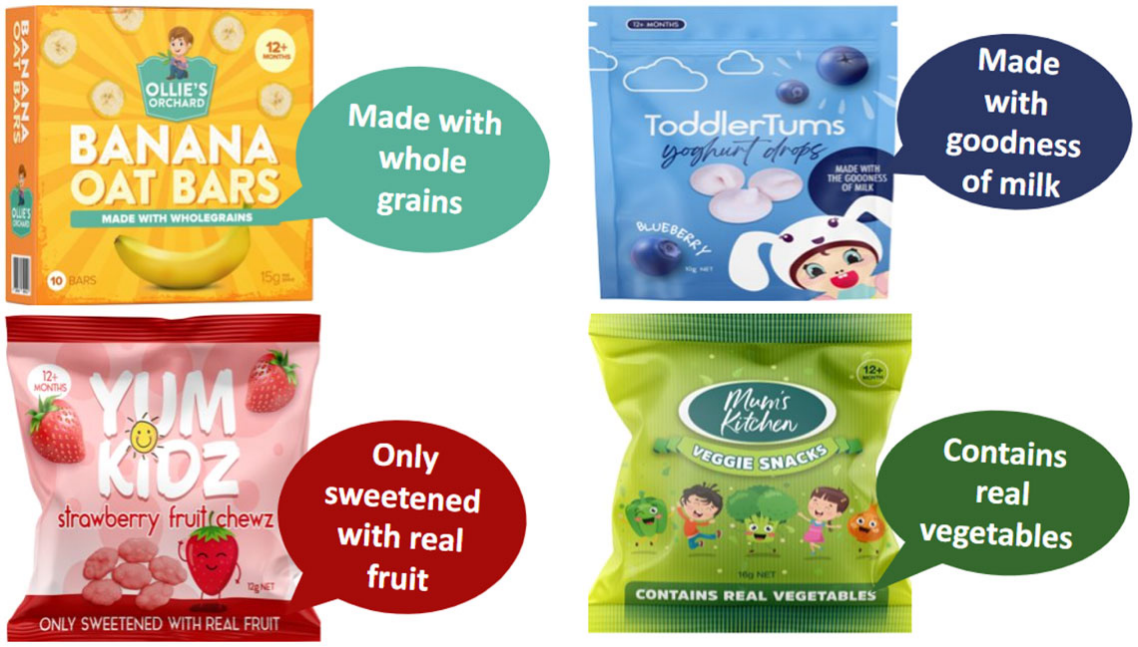	*“It feels like something a marketer would say, it feels a bit used‐car salesman, like you're saying all the right words, but it doesn't seem to hit as genuine.”*
	(G1, LE, co‐parent)
	*“It feels like it is just stating obvious.”*
	(G2, LE, co‐parent)
	*“Bit more factual in terms of it is made with whole grains so it is a bit more specific. It would definitely draw my attention, more than the other two [child‐related claims and ‘free from “bad” claims’ presented].”*
	(G3, LE, sole parent)
	*‘I don't know it just irritates me because ‘contains real vegetables’ well I'd hope that the veggie snacks don't contain fake veggies, I don't know, the phrases frustrate me.’*
	(G5, HE, coparent)
**Child‐related claims** 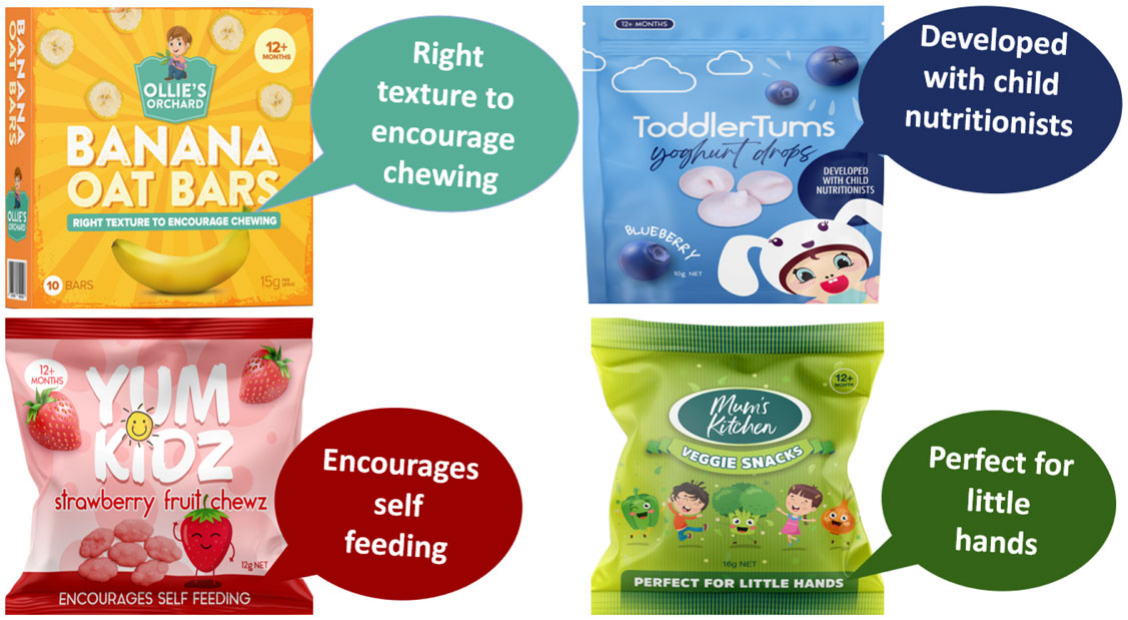	*“I think anyone, especially as a first‐time parent as well though those things can be helpful when you're unsure what to pick and if you're worried about choking and stuff when things aren't the right size or the right texture, that can make you feel more confident in picking that one rather than one that doesn't mention anything about it.”*
	(G2, LE, co‐parent)
	*I would be looking at those things from 6 months to say 8 to 9 months encouraging self‐feeding, chewing, all those things. Past 12 months, I think they have already got all those skills, I am not looking for those products anymore.*
	(G4, HE, co‐parent)

*Note:* *G = group.

### 2.3 Procedure

Focus groups were conducted via Zoom video conferencing in May 2023. The discussions were moderated by an experienced, qualitative researcher (KA) using the discussion guide and stimuli.

### 2.4 Data Analysis

Focus groups were audio‐recorded and transcribed verbatim. An exploratory content analysis approach was employed to thoroughly explore the data obtained from the discussion transcripts and identify key themes that emerged in response to the probing questions posed by the moderator. This approach involved a systematic examination of the transcripts, with a focus on uncovering patterns, relationships, and recurring ideas within the participants’ responses (Hsieh and Shannon 2005). The themes identified in the focus groups were then compared based on parental status and educational background to explore potential differences in themes related to these factors, as a previous survey found perceived healthiness and use of baby and toddler foods was higher among sole parents and parents with less education (The Royal Children's Hospital National Child Health Poll 2022). The analysis was conducted by a qualitative research expert (KA), who performed the initial coding and theme identification. To ensure robustness and reliability, the results were subsequently validated by a second researcher (MA) to enhance the credibility and accuracy of the findings. As no clear differences were identified based on participants’ parental status, educational level or gender, the results presented represent the overall prevailing response patterns across topics based on the stimuli, with specific quotes included for illustrative purposes. While this study focused on CTFs, we note that some respondents spoke collectively about commercial baby and toddler foods, suggesting that to these parents' similar issues apply to commercial baby foods as for CTFs.

### 2.5 Ethical Statement

Cancer Council Victoria Human Research Ethics Committee approved all procedures for this study (HREC 2228). Informed consent was obtained from all parents.

## 3 Results

### 3.1 Factors Influencing Parent's and Toddler's Choice of Toddler Foods

The initial discussion yielded insights into the product attributes and lifestyle considerations that parents and children pay attention to when evaluating and choosing toddler foods. Feeding toddlers a healthy diet was a high priority for all parents when choosing what to feed their child, although nutritional considerations became less important when calming a child, during travel or busy times, or when grandparents offered treats. Convenience was the primary reason given for purchasing CTFs over homemade or whole foods, particularly as travel snacks or quick meal options. While many parents repeatedly purchased familiar CTFs, they were also willing to try new products, particularly if they were on sale.

“First and foremost, for me, just looking at the nutritional content of the food.”

(Group 4 G4, high education (HE), co‐parent)

“Something that's healthy I can just chuck in the microwave, I don't have to cook anything, price.”

(G5, HE, coparent).

“Something that is convenient, I can grab if we are going to go out and something she can eat in the car as well.”

(G3, low education (LE), sole parent).

“I find myself switching sometimes whenever there are reasonable sales on something, so if haven't tried and it looks enticing and healthy and tasty and it is cheap, we definitely give it a go.”

(G4, HE, co‐parent).

When asked about the types of foods their toddler likes, parents mentioned homemade foods and whole foods such as fruits, rice and pasta, yet also indicated that their toddler ate CTFs daily. Key reasons parents gave for their toddler liking CTFs included visually appealing packaging (e.g., featuring bright colours or cartoons of popular children's TV characters), easy self‐feeding (e.g., hand held snacks that toddlers can eat independently), enjoyment from eating directly from the package (e.g., sucking on squeezy yoghurt pouches), and the toddler wanting the same food as their older siblings.

“She's obsessed with anything ‘Bluey’ [a cartoon puppy from a popular children's TV series] so if she sees the Bluey muesli bars, she would just run to it and grab it.”

(G2, LE, co‐parents).

### 3.2 Parents' Views on the Packaging and Labelling of CTFs

When shown images of CTFs without claims (Figure 1), parents felt the packaging's bright colours and characters were designed to appeal to parents and children, often associating brighter packaging with unhealthy food. Some expressed interest in trying healthier looking options like the banana oat bars or veggie snacks but said they would check the product's Nutrition Information Panel (NIP) first.

“They look like a fast snack …something that you can just grab off the shelf and they've got a healthy‐looking vibe to them…”

(G6, HE, sole parent).

### 3.3 Views on Claims on CTFs

Parents were shown CTFs displaying common claims and asked if they had seen these claims and if they found them appealing (see Table 1 for tested claims and illustrative quotes from parents). Parents were generally attracted to products displaying claims, though many expressed scepticism about the nutritional content of the product. There was high awareness of marketing tactics and lack of trust in claims. Parents expressed frustration with food companies marketing unhealthy products as healthy options and targeting time‐poor parents.

Parents had diverse views regarding different types of claims. Many found ‘free from “bad” ingredients’ claims slightly more appealing than other claim types tested, as they were considered to more clearly articulate the product was healthy. Some parents perceived ‘contains “good” ingredients’ claims as either self‐evident or eliciting suspicions and questions. For instance, when packs featured ‘made with real fruit’ claims, parents questioned what the product would consist of if not real fruit. Responses to child‐related claims were mixed, with some finding them appealing and innovative and others viewing them as irrelevant.

When asked why food companies use claims, parents generally viewed them as a strategic marketing tool to sell products and gain a competitive edge by positioning their products as superior to other brands and tapping into parental desires to do best for their children.

“I think that is why we are cynical because it is not always right, and it is great that we check the back of packets but there is a lot of parents out there that don't and that is a real disservice to the kids that they are marketing it like that, because if it has got something good written on the front then it should actually be good.”

(G3, LE, sole parent).

However, many parents were sceptical, seeing them as deceptive tactics designed to ‘trick people’. When asked who the claims were appealing to, parents mentioned that parents who are time‐poor, or health‐conscious would be more likely to be influenced by them.

“For time poor parents that are going to be easily influenced by those few key words [claims] on the front of the package, you are judging a book by its front cover, literally.” (G4, HE, co‐parent). “They are probably trying to appeal to more the healthy parents that are looking for better foods for their child.”

(G5, HE, co‐parent)

After parents were informed about our previous experimental study findings which showed that claims, particularly ‘free from “bad” ingredients’ claims, can boost parent's preferences, perceptions and purchasing intentions towards unhealthy CTFs (Dixon et al. 2024), most parents spontaneously noted that food companies used misleading marketing tactics.

### 3.4 Views on Regulating Claims on CTFs

Parents generally agreed there should be rules governing the use of claims, as well as other features of the labelling of CTFs such as product names, ingredients lists and nutritional information. They expressed concerns that current labelling practices, including the use of claims, do not provide sufficient clarity, making it difficult for parents to make informed decisions. There was strong agreement on the need for clear and honest labelling on CTFs, particularly regarding key nutritional information such as sugar content, to ensure that product names and on‐pack claims accurately reflect the product's ingredients and overall nutritional profile. Most parents believe companies should be legally required to provide accurate and truthful information on product packaging and that there should be rules around the nutrient composition of CTFs, showing support for introducing stricter regulation of claims. However, some parents voiced concerns that even with tighter regulations, companies would find alternative ways to market their products in ways that could still mislead parents. See illustrative quotes below.

“Absolutely, so they should (have restrictions on claims). Why are they able to put claims that aren't real?”

(G3, LE, co‐parent).

“I think it's a bit of a trap though as well with regulation, you're always going to get a team of lawyers who can come up with a statement which achieves much the same aims and gets around the regulations, so it's just a spiral, and I don't know that you can fix the problem.”

(G6, HE, sole parent)

During these discussions, it was apparent that many parents perceive a mismatch between the claims on the packet and nutritional profile of the product within. For example, parents mentioned concerns about claims that promote products as “healthy” despite containing high s, = levels of sugar. The strongest concerns regarding the nutritional profile of CTFs related to sugar, with many parents supporting restrictions on the amount of sugar permitted in CTFs. Parents viewed CTFs with high sugar levels as particularly harmful and expressed a desire for clearer FOP signposting of sugar in CTFs. Some parents also suggested that salt levels should be restricted or signposted on the FOP, to help parents identify CTFs that are lower in nutrients of concern.

“I'm a bit torn because I do think there's a really large uneducated amount of parents that would believe all of that and I think that's really sad because those are the kids that are going to end up with awful bad health, so in that sense I think yes put restrictions on claims but I also know that the companies will find a way around those.”

(G1, LE, co‐parent).

“I think it is incredibly important because if you look at the long‐lasting health implications, it ends up costing the society a lot of money with dealing with obesity and childhood behaviour issues and a lot of other things. I think from a government perspective, if they are going to actually start regulating the sugar industry and putting that information on, it may start helping parents whose children become so addicted to the taste of high sugar from such an early age.”

(G4, HE, co‐parent).

### 3.5 Information Sources Used for Guidance on Feeding Their Toddler a Healthy Diet

Parents drew on various information sources for guidance on how to feed their toddler a healthy diet, with information presented on product packaging or disseminated via social media emerging as valued and commonly used sources. In terms of on‐pack information, parents reported reviewing the ingredients list, using NIPs to compare sugar levels between products, checking use by dates, looking at Health Star Ratings (a voluntary FOP interpretive nutrition labelling scheme used in Australia and New Zealand [Shahid et al. 2020]) and relying on brand names that they perceive to be healthy. However, many parents struggled with understanding NIPs, interpreting serving sizes and determining acceptable levels of sugar and salt in CTFs. Many parents suggested that simple FOP nutrition labelling on CTFs would make it easier to compare the nutritional profile of different products of different CTFs.

“You want something that is meaningful that is easy to identify instead of having to look at a table of contents and ingredients to see which is the healthiest option”.

(G5, HE, co‐parent).

While child nutritionists and paediatricians were considered the most trustworthy sources of guidance on toddler feeding, many parents relied on social media (e.g., parent Facebook groups, Instagram, and TikTok) for recipe ideas and product recommendations for toddler meals and snacks. Some parents also used apps that provided information on food contents or suggested healthier alternatives when the product's bar code was scanned or relied on trusted parent groups and friends who had firsthand experience. The Maternal and Child Health Nurse was not deemed particularly helpful due to information being too generic and not addressing particular CTF products.

“Instagram, Facebook groups and Mum's Recommend, they've done their conversions, and they post all of the info and I'm like sweet.”

(G5, HE, co‐parent).

“I follow a couple of pediatric nutritionists on Instagram, and they'll often have different options or ideas or swaps for different foods…I definitely still get ideas from Instagram pages and stuff about things that are good and also they'll often do rundowns of new products.”

(G2, LE, co‐parent).

## 4 Discussion

This qualitative study provides insights into how claims on CTFs influence parents' product perceptions and preferences, assesses their views on such marketing, and examines their support for regulatory reform regarding the use of these claims. Parents generally found claims appealing, despite awareness of their persuasive intent and some mistrust of them. Parents want clear and honest labelling on CTFs including sugar content in CTFs, whereby product names and on‐pack claims accurately represent a product's ingredients and overall nutritional profile, including sugar content, while supporting stricter limits and clearer signposting to highlight potential harm. Many find NIPs difficult to interpret and often rely on social media for general and product‐specific advice on what to choose and feed their toddler. There was also clear support for stricter regulation of claims on CTFs, despite some parents raising concerns that industry would simply find another way to market their products.

Parents in this study prioritised choosing what they believe to be healthy CTFs for their toddlers, guided by NIPs and brand perceptions. However, there is a discrepancy between parental expectation and the actual nutritional profile of these products. Despite parents' best efforts to identify and provide healthy CTFs for their toddlers, most (82%) CTFs available in Australian supermarkets do not meet international all nutrition standards as per the WHO's Europe Nutrient and Promotion Profile Model (NPPM) (Scully et al. 2024) and that many contain high levels of added sugar (Scully et al. 2023). All CTFs feature on‐pack claims not permitted under the NPPM (Dunford et al. 2024; Scully et al. 2024). There is an urgent need for stricter regulations to better align Australian CTFs with international best practice for both nutritional profile and on‐pack claims. This would better align with public health goals and support the health and development of young children.

Convenience was cited as a key reason for purchasing CTFs. Foods that toddlers can eat directly from the packaging (such as pouches or finger foods) were considered especially convenient and ‘mess free’. An earlier product audit found that convenient packaging (e.g., squeeze pouches, single‐serve packets) is prevalent among commercial infant and toddler foods and emphasised through convenience‐focused messaging on the FOP (Chung et al. 2023), suggesting that food companies capitalise on parental concerns surrounding convenience. Prior research also consistently highlights convenience as a key parental consideration when choosing infant and toddler foods among Australian parents (Chung et al. 2023; The Royal Children's Hospital National Child Health Poll 2022), and UK parents (Hollinrake et al. 2024; Neve et al. 2024), with the latter studies also highlighting lack of time, insufficient support from partners, brand trust and nutrition content claims leading parents toward commercial foods.

Despite parents ‘awareness of and scepticism towards claims, they were still more attracted to products featuring these claims, especially ‘free from “bad” ingredients’ claims. This aligns with our previous study finding that ‘free from “bad” ingredients’ claims have a significant influence on parents' product preferences and purchase intentions (Dixon et al. 2024). This effect supports psychological theories suggesting that, claims highlighting the absence of negative ingredients appeal to risk‐avoidance motives and enhance perceived health benefits (Choi et al. 2013). These findings underscore the need for regulatory measures to ensure CTFs are labelled and marketed suitably and in a manner that accurately reflects the overall nutritional content of the product.

This study found that while many parents perceived ‘contains “good” ingredients’ claims as either self‐evident or suspicious, some were drawn to specific examples of such claims (e.g., ‘made with whole grains’). These claims often engender a positive emotional response in parents, creating a perception of nutritional benefits that may not be entirely warranted by the product's overall nutritional profile. This effect may be attributed to the psychological tendency known as the ‘health halo,’ where claims about particular product attributes enhance the overall perception of a product's healthiness, overshadowing unhealthy nutritional content, as supported by previous research (Hall et al. 2022; Harris et al. 2011; Simmonds et al. 2021; Velázquez et al. 2021). Our previous study also showed that parents with three or more children (who may be especially time poor) were more likely to select unhealthy CTFs if they displayed ‘contains “good” ingredient’ claims (cf. no claim) (Dixon et al. 2024). Another direct choice experiment found that nutrition content claims influenced parents' perceptions of the healthiness of unhealthy CTFs and milks, and that parents were nearly twice as likely to choose a product with the claim ‘made with real fruit’ (McCann et al. 2022). Parents, particularly time poor parents, may rely on these claims to guide their food choices, leading them to inadvertently select products that do not align with their health goals. This highlights the need for more transparent and accurate marketing practices that genuinely reflect a product's nutritional value, thereby supporting better informed choices by parents.

While ‘child‐related claims’ were appealing to some parents, others viewed them as irrelevant. In our previous experiment, child‐related claims did not influence most parents' product choice, purchasing intentions, or perceptions, except among sole parents who showed higher purchase intentions for unhealthy products displaying these claims (Dixon et al. 2024). To date, few studies have assessed effects of such claims on consumers (McCann et al. 2022).

Parents spontaneously mentioned how visual elements of packaging such as bright colours and the depiction of cartoon characters boost the appeal of certain CTFs to their toddler, which in turn influences parent's food choices for their children. Similarly, previous research has found that colourful designs (Letona et al. 2014) and licensed characters (Velázquez et al. 2021) on packaging influence children's food preferences and parent's purchasing intentions. Given most toddlers are preliterate, it makes sense that visual rather than textual elements of CTFs appeal to them. A recent audit in major Australian supermarkets found that 9 out of 10 infant and toddler food packages displayed child‐appealing marketing techniques, which were dominated by visual elements such as bright colours, graphics, cartoons, branded characters, and images of a baby or young child on FOP (Chung et al. 2023). Food companies use child‐appealing features to attract the attention of young children and additional marketing techniques like claims to convey to parents that their products are healthy and desirable, creating the overall impression that these products are good for young children (Lapierre et al. 2017). As many of these products are highly processed, contain high amounts of sugar and are not nutritionally adequate (Dunford et al. 2024; Scully et al. 2024; Scully et al. 2023), we concur with Chung et al.'s (2023) recommendation, that “government‐led controls on all marketing intended to influence children's diets are necessary to adequately protect young children from the harmful impact of food marketing and to promote the development of optimal feeding behaviours. Specifically, in the context of marketing infant and toddler foods, this includes the regulation of marketing techniques including claims targeted at both children and their caregivers” (pp 1) (Chung et al. 2023).

Parents in the current study showed a tendency to be swayed towards new products when they were on sale. A recent content analysis found that the majority of commercial baby and toddler foods shown in Australian supermarket catalogues were accompanied by price‐related promotions (Chung A et al. 2024), and other audits of supermarket catalogues have found that price promotions are more common on unhealthy foods (Grigsby‐Duffy et al. 2020; Riesenberg et al. 2019). The findings from prior research and the present study indicate that the government should consider prohibiting price promotions on unhealthy CTFs to mitigate their influence on parental purchasing decisions and promote healthier food choices for children.

While many parents showed support for stricter regulation of claims, some raised concerns about whether stronger regulation would be effective. These concerns stemmed from a perception that industry would find artful ways to circumvent rules and promote their products using other techniques. Consumer laws already require that food companies provide accurate information on product packaging. However, although being technically true, these claims can still mislead parents about the overall nutritional content of CTFs. Regulations should be introduced in line with WHO recommendations to ensure these foods are free from all claims as this would better support the health and development of toddlers. The strong parental support for restrictions on sugar content echoes a survey of Australian parents, which found 90% agreed that there should be laws to restrict sugar and salt in baby and toddler foods (The Royal Children's Hospital National Child Health Poll 2022). Additionally, the present study found that parents expect clear, truthful labelling and support laws about the words, images and claims that can be used on the packaging of baby and toddler foods, including disclosure of sugar. Research indicates that sugar warning labels offer a promising strategy, informing consumers and promoting healthier food choices, including on sugary drinks (Miller et al. 2022; Scapin et al. 2021; Taillie et al. 2022).

This study also revealed that many parents rely on social media platforms for nutrition information about what to feed their toddler, rather than on mandatory nutrient information (NIP and ingredients list) or face to face guidance from health professionals. This phenomenon appeared to be driven by parents perceiving that the information presented in social media is highly accessible and understandable, whereas mandatory nutrition information (NIP and ingredients list) can be difficult to interpret, and the nutrition ‘experts’ appearing on social media were more likely to review specific products than their maternal and child health nurse, who only provides generic feeding advice. Previous studies (De Rosso et al. 2022; Frey et al. 2022) have shown that parents are motivated to use social media as a source of health information and for making decisions about feeding their young children. However, caution is needed, as influential social media bloggers often receive incentives from brands to promote their products and are not nutritionists or health professionals. For instance, TikTok has emerged as a source of unhealthy food marketing, including content created by users under the influence of brands (Brooks et al. 2022; Minadeo and Pope 2022), leveraging TikTok users as ‘unofficial brand ambassadors,’ thereby exerting influence on consumers. This highlights the need for actions to address marketing unhealthy CTFs via digital media. Additionally, government, public health organisations and reputable child nutrition experts could utilise social media platforms to reach and engage parents with accessible, practical, evidence‐based guidance concerning optimal early childhood nutrition.

### 4.1 Strengths and Limitations

This study provides in‐depth insights into parent's perceptions and preferences regarding claims and regulations for claims in relation to CTFs. The focus group discussions were facilitated by an independent research consultant who is highly experienced in qualitative studies and behavioural science. However, several limitations should be noted. Firstly, findings may not be generalisable to the broader population since the research was conducted in a single state and relied on a small convenience sample. Second, the information gathered primarily reflects the experiences and perceptions of the participating parents and may not be representative of all parents' views. Third, we used digital images of mock CTFs to illustrate common claim types, which could have meant participant's responses diverged from how they would react to claims on real products in naturalistic food retail settings. However, the strength of this approach was that the mock products, which were professionally designed, removed the issue of prior brand knowledge or attitudes confounding responses to the claims. Finally, this study explored single claims on CTFs, which may not have as strong an effect as when multiple claims are displayed on a single product, as is common in Australia (Dunford et al. 2024; Scully et al. 2024).

### 4.2 Policy Implications

The use of claims on commercial infant and toddler foods is becoming increasingly common in Australia (McCann et al. 2021a), products typically have multiple claims with products carrying eight claims on average and up to 20 on a single packet (Scully et al. 2024). Concerningly, these claims can create a ‘health halo’ over products and nudge parents towards choosing foods that tend to have a sub‐optimal nutrition profile, be ultra‐processed, and bear little resemblance to nutritious core foods that authorities recommended toddlers eat for lifelong health. There is a need for the government to regulate claims, ensuring that commercial infant and toddler foods display product names that accurately reflect ingredients and *do not* display any claims on food packaging in accordance with WHO Europe's NPPM recommendations (World Health Organization Regional Office for Europe 2022) and protecting them from the influence of harmful marketing practices of the food industry. Marketing strategies using bright colours and characters can appeal to parents and children, influencing purchasing preferences. Parents also increasingly rely on social media for nutrition information, making it essential to regulate digital marketing. There is also strong parental support for restrictions on sugar content and honest labelling regarding sugar content, highlighting the need for policies that focus on transparent sugar labelling.

## 5 Conclusions

This study provides valuable insights into parents' perspectives on CTFs and the claims that commonly appear on these products. Despite parents' awareness and scepticism regarding these claims, they remain influenced by them. Parents call for the regulation of claims on CTFs, with strong support for regulating sugar content in these products and ensuring transparent labelling, including clear disclosure of sugar content. Findings from this study align with broader recommendations for improved regulation of the marketing and labelling of child‐targeted food products, to protect children's health. Parents agree there should be laws about the words, images and claims on CTFs. As noted by Chung et al. (2023), such controls are essential to protect young children from the impact of food marketing and to promote healthier food choices. Specifically, regulation of toddler food marketing techniques, including those targeting both children and their caregivers, is crucial. Study findings support calls for stricter regulations to limit claims on CTFs.

## Author Contributions

H.D. conceptualised and designed the study, with input from all co‐authors in refining the study protocols. K.A. facilitated the focus groups, qualitatively analysed the data, and conducted the initial interpretation and reporting. M.A. coordinated the project and prepared the initial draft of the paper. M.A., M.S., A.S. and H.D. interpreted the results, edited, and finalised the manuscript, with substantial input from all authors. All authors have checked and approved the submitted manuscript.

## Conflicts of Interest

The authors declare no conflicts of interest. Most of the authors (M.A., M.S., A.S., A.M., J.M. and H.D.) work for an organisation that conducts public health interventions, research, and advocacy aimed at reducing diet related health risks, obesity, and cancer.

## Supporting information

Supplementary Information

## Data Availability

The data that support the findings of this study are available on request from the corresponding author. The data are not publicly available due to privacy or ethical restrictions.

## References

[mcn70032-bib-0001] Birch, L. , J. S. Savage , and A. Ventura . 2007. “Influences on the Development of Children's Eating Behaviours: From Infancy to Adolescence.” Canadian Journal of Dietetic Practice and Research 68, no. 1: s1–s56. 19430591 PMC2678872

[mcn70032-bib-0002] Brooks, R. , R. Christidis , N. Carah , B. Kelly , F. Martino , and K. Backholer . 2022. “Turning Users Into ‘Unofficial Brand Ambassadors’: Marketing of Unhealthy Food and Non‐Alcoholic Beverages on TikTok.” BMJ Global Health 7, no. 6: e009112. 10.1136/bmjgh-2022-009112. PMC9240823 35879104

[mcn70032-bib-0003] Chang, K. , N. Khandpur , D. Neri , et al. 2021. “Association Between Childhood Consumption of Ultraprocessed Food and Adiposity Trajectories in the Avon Longitudinal Study of Parents and Children Birth Cohort.” JAMA Pediatrics 175, no. 9: e211573. 10.1001/jamapediatrics.2021.1573. 34125152 PMC8424476

[mcn70032-bib-0004] Chen, X. , Z. Zhang , H. Yang , et al. 2020. “Consumption of Ultra‐Processed Foods and Health Outcomes: A Systematic Review of Epidemiological Studies.” Nutrition Journal 19, no. 1: 86. 10.1186/s12937-020-00604-1. 32819372 PMC7441617

[mcn70032-bib-0005] Choi, H. , K. Yoo , T. Hyun Baek , L. N. Reid , and W. Macias . 2013. “Presence and Effects of Health and Nutrition‐Related (HNR) Claims With Benefit‐Seeking and Risk‐Avoidance Appeals in Female‐Orientated Magazine Food Advertisements.” International Journal of Advertising 32, no. 4: 587–616. 10.2501/IJA-32-4-587-616.

[mcn70032-bib-0006] Chung, A. , J. Myers , H. Skouteris , and K. Backholer . 2023. “Front‐of‐Pack Marketing on Infant and Toddler Foods: Targeting Children and Their Caregivers.” Australian and New Zealand Journal of Public Health 47, no. 6: 100101. 10.1016/j.anzjph.2023.100101. 38030442

[mcn70032-bib-0007] Chung, A. T. S. , H. Dixon , J. McCann , A. Schmidtke , and C. Fleming . 2024. “Infant and Toddler Food Marketing in Supermarket Catalogues: Promoting Health or Misinformation?” Australian and New Zealand Journal of Public Health, Under review. 10.1093/heapro/daaf043.

[mcn70032-bib-0008] Dixon, H. , M. Scully , M. Wakefield , B. Kelly , K. Chapman , and R. Donovan . 2011. “Parent's Responses to Nutrient Claims and Sports Celebrity Endorsements on Energy‐Dense and Nutrient‐Poor Foods: An Experimental Study.” Public Health Nutrition 14, no. 6: 1071–1079. 10.1017/S1368980010003691. 21306666

[mcn70032-bib-0009] Dixon, H. G. , M. A. Awoke , M. Scully , et al. 2024. “Effects of Marketing Claims on Toddler Food Products on parents' Product Preferences, Perceptions and Purchasing Intentions: An Online Experiment.” International Journal of Behavioral Nutrition and Physical Activity 21, no. 1: 60. https://ijbnpa.biomedcentral.com/counter/pdf/10.1186/s12966-024-01603-9.pdf. 38773586 10.1186/s12966-024-01603-9 PMC11110258

[mcn70032-bib-0010] Dunford, E. K. , M. Scully , and D. Coyle . 2024. “Commercially‐Produced Infant and Toddler Foods—How Healthy Are They? An Evaluation of Products Sold in Australian Supermarkets.” Maternal & Child Nutrition 20: e13709. 39133124 10.1111/mcn.13709 PMC11574637

[mcn70032-bib-0011] Fleming‐Milici, F. , L. Phaneuf , and J. L. Harris . 2022. “Marketing of Sugar‐Sweetened Children's Drinks and parents' Misperceptions About Benefits for Young Children.” Maternal & Child Nutrition 18: e13338. 10.1111/mcn.13338. 35199914 PMC9218304

[mcn70032-bib-0012] Food Standards Australia New Zealand . 2013. Australia New Zealand Food Standards Code ‐ Standard 1.2.7 ‐ Nutrition, Health and Related Claims. Commonwealth of Australia. Retrieved 7 October from http://www.comlaw.gov.au/Details/F2013L00054.

[mcn70032-bib-0013] Frey, E. , C. Bonfiglioli , M. Brunner , and J. Frawley . 2022. “parents' Use of Social Media as a Health Information Source for Their Children: A Scoping Review.” Academic Pediatrics 22, no. 4: 526–539. 10.1016/j.acap.2021.12.006. 34906742

[mcn70032-bib-0014] García, A. L. , G. Morillo‐Santander , A. Parrett , and A. N. Mutoro . 2019. “Confused Health and Nutrition Claims in Food Marketing to Children Could Adversely Affect Food Choice and Increase Risk of Obesity.” Archives of Disease in Childhood 104, no. 6: 541–546. 10.1136/archdischild-2018-315870. 30530844

[mcn70032-bib-0015] Grigsby‐Duffy, L. , S. Schultz , L. Orellana , et al. 2020. “The Healthiness of Food and Beverages on Price Promotion at Promotional Displays: A Cross‐Sectional Audit of Australian Supermarkets.” International Journal of Environmental Research and Public Health 17, no. 23: 9026. 10.3390/ijerph17239026. 33287395 PMC7729449

[mcn70032-bib-0016] Hall, M. G. , A. J. Lazard , I. C. Higgins , et al. 2022. “Nutrition‐Related Claims Lead Parents to Choose Less Healthy Drinks for Young Children: A Randomized Trial in a Virtual Convenience Store.” American Journal of Clinical Nutrition 115, no. 4: 1144–1154. 10.1093/ajcn/nqac008. 35040866 PMC8971006

[mcn70032-bib-0017] Harris, J. L. , J. M. Thompson , M. B. Schwartz , and K. D. Brownell . 2011. “Nutrition‐Related Claims on Children's Cereals: What Do They Mean to Parents and Do They Influence Willingness to Buy?” Public Health Nutrition 14, no. 12: 2207–2212. 10.1017/S1368980011001741. 21806872

[mcn70032-bib-0018] Hoare, A. , M. Virgo‐Milton , R. Boak , et al. 2014. “A Qualitative Study of the Factors That Influence Mothers When Choosing Drinks for Their Young Children.” BMC Research Notes 7: 430. 10.1186/1756-0500-7-430. 24997015 PMC4097085

[mcn70032-bib-0019] Hollinrake, G. , S. Komninou , and A. Brown . 2024. “Use of Baby Food Products During the Complementary Feeding Period: What Factors Drive parents' Choice of Products?” Maternal & Child Nutrition 20: e13689. 38898599 10.1111/mcn.13689 PMC11574642

[mcn70032-bib-0020] Hsieh, H. F. , and S. E. Shannon . 2005. “Three Approaches to Qualitative Content Analysis.” Qualitative Health Research 15, no. 9: 1277–1288. 10.1177/1049732305276687. 16204405

[mcn70032-bib-0021] Hu, F. B. 2013. “Resolved: There Is Sufficient Scientific Evidence That Decreasing Sugar‐Sweetened Beverage Consumption Will Reduce the Prevalence of Obesity and Obesity‐Related Diseases.” Obesity Reviews 14, no. 8: 606–619. 10.1111/obr.12040. 23763695 PMC5325726

[mcn70032-bib-0022] Isaacs, A. , K. Neve , and C. Hawkes . 2022. “Why Do Parents Use Packaged Infant Foods When Starting Complementary Feeding? Findings From Phase One of a Longitudinal Qualitative Study.” BMC Public Health 22, no. 1: 2328. 10.1186/s12889-022-14637-0. 36510175 PMC9744586

[mcn70032-bib-0023] Lapierre, M. A. , A. M. Brown , H. V. Houtzer , and T. J. Thomas . 2017. “Child‐Directed and Nutrition‐Focused Marketing Cues on Food Packaging: Links to Nutritional Content.” Public Health Nutrition 20, no. 5: 765–773. 10.1017/s1368980016002317. 27609415 PMC10261630

[mcn70032-bib-0024] Leffa, P. S. , D. J. Hoffman , F. Rauber , C. N. Sangalli , J. L. Valmórbida , and M. R. Vitolo . 2020. “Longitudinal Associations Between Ultra‐Processed Foods and Blood Lipids in Childhood.” British Journal of Nutrition 124, no. 3: 341–348. 10.1017/S0007114520001233. 32248849

[mcn70032-bib-0025] Letona, P. , V. Chacon , C. Roberto , and J. Barnoya . 2014. “A Qualitative Study of Children's Snack Food Packaging Perceptions and Preferences.” BMC Public Health 14: 1274. 10.1186/1471-2458-14-1274. 25511663 PMC4300846

[mcn70032-bib-0026] Maubach, N. , and J. Hoek . 2010. “A Qualitative Study of New Zealand parents' Views on Front‐of‐Pack Nutrition Labels.” Nutrition & Dietetics 67, no. 2: 90–96. 10.1111/j.1747-0080.2010.01425.x.

[mcn70032-bib-0027] McCann, J. , J. Woods , M. Mohebbi , and C. G. Russell . 2022. “Regulated Nutrition Claims Increase Perceived Healthiness of an Ultra‐Processed, Discretionary Toddler Snack Food and Ultra‐Processed Toddler Milks: A Discrete Choice Experiment.” Appetite 174: 106044. 10.1016/j.appet.2022.106044. 35430297

[mcn70032-bib-0028] McCann, J. R. , C. G. Russell , and J. L. Woods . 2021a. “The Nutritional Profile and On‐Pack Marketing of Toddler‐Specific Food Products Launched in Australia Between 1996 and 2020.” Nutrients 14, no. 1: 163. 10.3390/nu14010163. 35011037 PMC8747045

[mcn70032-bib-0029] McCann, J. R. , C. G. Russell , K. J. Campbell , and J. L. Woods . 2021b. “Nutrition and Packaging Characteristics of Toddler Foods and Milks in Australia.” Public Health Nutrition 24, no. 5: 1153–1165. 33183396 10.1017/S1368980020004590 PMC10195549

[mcn70032-bib-0030] Miller, C. , K. Ettridge , S. Pettigrew , et al. 2022. “Warning Labels and Interpretive Nutrition Labels: Impact on Substitution Between Sugar and Artificially Sweetened Beverages, Juice and Water in a Real‐World Selection Task.” Appetite 169: 105818. 10.1016/j.appet.2021.105818. 34838869

[mcn70032-bib-0031] Minadeo, M. , and L. Pope . 2022. “Weight‐Normative Messaging Predominates on TikTok—A Qualitative Content Analysis.” PLoS One 17, no. 11: e0267997. 10.1371/journal.pone.0267997. 36318532 PMC9624392

[mcn70032-bib-0032] Monteiro, C. A. , G. Cannon , R. B. Levy , et al. 2019. “Ultra‐Processed Foods: What They Are and How to Identify Them.” Public Health Nutrition 22, no. 5: 936–941. 10.1017/S1368980018003762. 30744710 PMC10260459

[mcn70032-bib-0033] National Health and Medical Research Council . (2003). Dietary Guidelines for Children and Adolescents in Australia. Australian Government.

[mcn70032-bib-0034] Neri, D. , E. M. Steele , N. Khandpur , et al. 2022. “Ultraprocessed Food Consumption and Dietary Nutrient Profiles Associated With Obesity: A Multicountry Study of Children and Adolescents.” Supplement, Obesity Reviews: An Official Journal of the International Association for the Study of Obesity 23, no. S1: e13387. 10.1111/obr.13387. 34889015

[mcn70032-bib-0035] Neve, K. L. , P. Coleman , C. Hawkes , C. Vogel , and A. Isaacs . 2024. “What Shapes Parental Feeding Decisions Over the First 18 Months of Parenting: Insights Into Drivers Towards Commercial and Home‐Prepared Foods Among Different Socioeconomic Groups in the UK.” Appetite 196: 107260. 38403201 10.1016/j.appet.2024.107260

[mcn70032-bib-0036] Pries, A. M. , E. Bassetti , J. Badham , et al. 2024. “Ultraprocessing and Presence of Additives in Commercially Produced Complementary Foods in Seven Southeast Asian Countries: A Cross‐Sectional Study.” The American Journal of Clinical Nutrition 120, no. 2: 310–319. 10.1016/j.ajcnut.2024.04.003. 38816268 PMC11347792

[mcn70032-bib-0037] Ravikumar, D. , E. Spyreli , J. Woodside , M. McKinley , and C. Kelly . 2022. “Parental Perceptions of the Food Environment and Their Influence on Food Decisions Among Low‐Income Families: A Rapid Review of Qualitative Evidence.” BMC Public Health 22, no. 1: 9. 10.1186/s12889-021-12414-z. 34983469 PMC8727174

[mcn70032-bib-0038] Richter, A. P. C. , E. W. Duffy , L. Smith Taillie , J. L. Harris , J. L. Pomeranz , and M. G. Hall . 2022. “The Impact of Toddler Milk Claims on Beliefs and Misperceptions: A Randomized Experiment With Parents of Young Children.” Journal of the Academy of Nutrition and Dietetics 122, no. 3: 533–540.e3. 10.1016/j.jand.2021.08.101. 34391941 PMC8840993

[mcn70032-bib-0039] Riesenberg, D. , K. Backholer , C. Zorbas , et al. 2019. “Price Promotions by Food Category and Product Healthiness in an Australian Supermarket Chain, 2017–2018.” American Journal of Public Health 109, no. 10: 1434–1439. 10.2105/ajph.2019.305229. 31415196 PMC6727276

[mcn70032-bib-0040] De Rosso, S. , S. Nicklaus , P. Ducrot , and C. Schwartz . 2022. “Information Seeking of French Parents Regarding Infant and Young Child Feeding: Practices, Needs and Determinants.” Public Health Nutrition 25, no. 4: 879–892. 10.1017/S1368980021003086. 34321131 PMC9991613

[mcn70032-bib-0041] Scaglioni, S. , V. De Cosmi , V. Ciappolino , F. Parazzini , P. Brambilla , and C. Agostoni . 2018. “Factors Influencing Children's Eating Behaviours.” Nutrients 10, no. 6: 706. 10.3390/nu10060706. 29857549 PMC6024598

[mcn70032-bib-0042] Scapin, T. , A. C. Fernandes , C. C. Curioni , et al. 2021. “Influence of Sugar Label Formats on Consumer Understanding and Amount of Sugar in Food Choices: A Systematic Review and Meta‐Analyses.” Nutrition Reviews 79, no. 7: 788–801. 10.1093/nutrit/nuaa108. 33313917

[mcn70032-bib-0043] Scully, M. , R. Jinnette , L. Le , J. Martin , and A. Schmidtke . 2024. “Compliance of Australian Commercial Foods for Young Children (<36 Months) With an International Nutrient and Promotion Profile Model.” Australian and New Zealand Journal of Public Health 48, no. 3: 100158. 10.1016/j.anzjph.2024.100158. 38886145

[mcn70032-bib-0044] Scully, M. , A. Schmidtke , L. Conquest , J. Martin , and A. McAleese . 2023. “Commercially Available Foods for Young Children (<36 Months) in Australia: An Assessment of How They Compare to a Proposed Nutrient Profile Model.” Health Promotion Journal of Australia: Official Journal of Australian Association of Health Promotion Professionals 34: 750–758. 10.1002/hpja.705. 36786727

[mcn70032-bib-0045] Shahid, M. , B. Neal , and A. Jones . 2020. “Uptake of Australia's Health Star Rating System 2014‐2019.” Nutrients 12, no. 6: 1791. 10.3390/nu12061791. 32560224 PMC7353262

[mcn70032-bib-0046] Simmonds, L. , A. L. Brownbill , A. Zee , and M. J. Netting . 2021. “Health‐Related Marketing Messages on Product Labels of Commercial Infant and Toddler Food Packaging in Australia: A Cross‐Sectional Audit.” BMJ Paediatrics Open 5, no. 1: e001241. 10.1136/bmjpo-2021-001241.

[mcn70032-bib-0047] Taillie, L. S. , I. C. A. Higgins , A. J. Lazard , D. R. Miles , J. L. Blitstein , and M. G. Hall . 2022. “Do Sugar Warning Labels Influence parents' Selection of a Labeled Snack for Their Children? A Randomized Trial in a Virtual Convenience Store.” Appetite 175: 106059. 10.1016/j.appet.2022.106059. 35526703 PMC10173438

[mcn70032-bib-0048] The Royal Children's Hospital National Child Health Poll . (2022). “Ready‐Made Baby Foods: Do Parents Know the facts?” https://www.rchpoll.org.au/wp-content/uploads/2022/04/NCHP24-Poll-report-A4_FA_WEB.pdf.

[mcn70032-bib-0049] Velázquez, A. L. , F. Alcaire , L. Vidal , P. Varela , T. Næs , and G. Ares . 2021. “The Influence of Label Information on the Snacks Parents Choose for Their Children: Individual Differences in a Choice Based Conjoint Test.” Food Quality and Preference 94: 104296.

[mcn70032-bib-0050] Ventura, A. K. , and J. Worobey . 2013. “Early Influences on the Development of Food Preferences.” Current Biology 23, no. 9: R401–R408. 10.1016/j.cub.2013.02.037. 23660363

[mcn70032-bib-0051] World Health Organization Regional Office for Europe . (2022). Nutrient and Promotion Profile Model: Supporting Appropriate Promotion of Food Products for Infants and Young Children 6‐36 Months in the WHO European Region. World Health Organization.

