# Supplementary material for: Parents' Perceptions of Claims on Packaged Commercial Toddler Foods: A Qualitative Study
Source: Matern Child Nutr. 2025 Apr 22;21(3):e70032. doi: 10.1111/mcn.70032 (PMC12150125; doi:10.1111/mcn.70032)
Supplement: Supplementary file 1 — Supplementary Information [file MCN-21-e70032-s001.docx]

**Supp_ file 1**

**Focus Group Discussion Guide**

1. Introduction and sorting out any technical issues (10 mins)

Hello, my name is Kerryn Alexander, I’m an independent research consultant.

Welcome to our focus group about different types of toddler foods.

At the end of the discussion, I’ll tell you who the research is being conducted for.

Process

Check previous involvement in focus groups and explain process:

- Because we are using Zoom, we need to do things a bit differently than face to face focus groups. I’m sometimes going to ask each person for their view and other times would like to have a discussion among the group.
- It’s really important that everyone has the opportunity to speak.
- Please be careful not to speak over other people so that we can all hear the discussion.
- If you can, its best to mute yourself when you are not talking so that we don’t hear any background noise and please make sure you are in a quiet place without your children tv or music in the background.
- We are interested in hearing about the range of views people have about this topic. There are no right or wrong answers It is not necessary for everyone to agree with each other, so please speak up if your views are different from those of other people in the group.

Confidentiality

- Everything you say and your personal details are confidential.
- We will be recording the group. The video recording will be transcribed and will only be used to help us to accurately capture your comments so we can report on the range of responses. After the project is complete, the recording will be destroyed.
- I’m going to turn on the recording now, so can you each please confirm by saying ‘Yes’ (as you agreed during recruitment) that you are happy for us to record the group?
- Does anyone have any questions before we start?

1. Participant Introductions (5 mins [15])

To start with, I’d like to ask everyone to introduce themselves in turn and tell us:

(names already on zoom)

- How many children you have and their ages
- What you do during the day (work, study, caregiver etc)

1. Toddler food products and purchase behaviour (15 mins [30])

Now I’d like to hear about what sorts of foods **your toddler really likes**.

***Ask each in turn***

Prompts if necessary:

- - Pre-prepared toddler foods vs. home-prepared, whole, unprocessed foods
  - Attributes of the food itself (colour, flavour, sweetness, texture, shape of food, finger food, puree)
  - Attributes of the packaging/marketing (e.g., characters on the packet, colours of the packet, shape of the packet, eg squeezy pouch).

Now thinking more about **readymade toddler foods** that you can buy at the supermarket…

- Are there particular readymade toddler foods that you **buy** for your toddler?
- How often would your toddler eat these ready made foods?
- Why do you buy these particular readymade toddler foods? What things are important when choosing these foods?

Prompts if necessary:

- - Health/nutrition
  - Price
  - Convenience
  - Packaging
  - Flavour or food content
  - Labelling (front of pack)
  - Information panel (back of pack
  - Toddler’s request/preference
  - Allergies/dietary restrictions
  - Recommendations
  - Not messy/can eat in car

**If time allows:**

- Do you normally buy the same readymade foods for your toddler, or do you switch between different ones? Why?

1. Importance of health and nutrition in toddler food purchases (10 mins [40])

- When you’re choosing foods to buy for your toddler, how important is the healthiness or nutritional value of the food?
- Are there any situations where the health or nutritional value of the food is less important?
- How do you know whether a toddler food product is healthy or not?

Prompts if necessary:

- - Packaging
  - labelling (specific ingredients, allergens
  - marketing
- Do you look at the labels or nutritional panel on the back of the packet?
- What sort of information **do you look for** on the labels when you’re choosing something to buy or feed your toddler?

1. Labelling and marketing of toddler foods

Ready made foods without claims shown (5 mins [45])

Now we're going to talk about how toddler food products are packaged and marketed.

I’m going to show you a slide of some readymade toddler foods.

*Show Slide 1*

- What comes to mind when looking at these toddler food products?
- Who do you think these foods might appeal to (as purchaser and consumer)?

Examination of three types of claims (20 mins [65])

Now I’m going to show some examples of toddler foods with claims on the packaging.

*Show Slides 2, 3 and 4 randomised*

*Contains good ingredients claim*

*Free from bad ingredients claim*

*Unregulated child related claim*

I’d like you to focus on the **types of claims** that are shown on these food packs, without paying too much attention to the flavours or other features.

*Show first slide*

These toddler foods are labelled with claims such as:

*Read out claims on slide*

- In what ways do these claims **appeal** to parents and carers?

Prompts if necessary:

- - healthier choice for their toddler
  - contains good/healthy ingredients
  - free from bad/unhealthy ingredients
  - higher quality
  - Good for toddler’s growth/development
  - Won’t harm their toddler
- Have **you** **seen** toddler foods with claims like these?
- Have **you bought or would you buy** products with this type of feature over others when buying foods for your toddler? Why/Why not?
- **Who** do you think toddler food products with these types of claims would be appealing to? Do they appeal to people like you? If not, who?

*Repeat above question group for other stimulus slides*

*Explore any differences/similarities between perceptions of the different types of claims on each slide set.*

Most effective claim type (5 mins [70])

What type of claim do you think is the most effective? Why?

***Ask each in turn***

*Contains good ingredients claim*

*Free from bad ingredients claim*

*Unregulated child related claim*

Why do you think food companies put this type of information on toddler food products?

1. How these claims influence other parents (Study 1 findings) (5 mins) [75])

*Show Slide 5*

Toddler food products with these sorts of health-related ingredient claims, or child-related claims are not necessarily the best choices for your toddler. They may contain high amounts of sugar, which can cause tooth decay, unhealthy weight gain, and set toddlers up with a preference for sweet foods.

*Show slide 6*

The organisation conducting this research has found in previous research they have conducted that when marketing claims appear on unhealthy toddler products, they encourage some parents to:

- Think these products are healthy
- Think they are good foods for toddlers
- Prefer these products over healthier options
- Want to buy them for their toddler

Reflections and policy options (10 mins [85])

- How do you feel about food companies putting these sorts of claims on toddler foods?
- Some health groups would like restrictions on certain types of promotional claims on toddler foods, because of concerns that these claims nudge parents/carers towards buying products that aren’t necessarily the healthiest options for their toddlers.

Would you be supportive of this move? Why/why not?

- Did you know that there are currently no specific regulations about what can and cannot be put into foods for toddlers?

Do you think there should be? For which ingredients?

- Some health groups would like to see limits on how much sugar, including processed fruit sugar, can be added to foods for toddlers. Would you be supportive of this??

Why/why not?

- It is currently difficult for parents to identify added sugar in a product as the label only shows total sugar (total sugar includes added sugars as well as the sugars that are naturally occurring in dairy foods and whole fruit and vegetables).

Some health groups would like to see clear added sugar (including processed fruit sugar) labelling on food labels.

Would you be supportive of this? Why/why not?IF TIME ALLOWS

1. Information needs (10 mins [85])

- Do you look for information about how to feed your toddler a healthy diet?
- Where do you find that information?
- What do you think are the most trustworthy sources of information about healthy eating for toddlers?

1. Thank you and close (5 minutes [90])

Thank you for a very helpful and interesting discussion.

I told you at the beginning that I would let you know who the research is being conducted for:

“This research is being conducted on behalf of Cancer Council Victoria who are interested in better understanding parent’s and carer’s views on readymade toddler food products that feature claims about nutrition content, health-related ingredients or product benefits to children.

The results will be used to inform future education resources for parents about toddler foods, plus advocacy urging government and industry to improve the marketing and labelling of toddler food products to make it easier for parents to identify the healthiest options for their children.”

We have some further information about dietary guidelines and reading food labels that can be emailed to you if you are interested.

***Table 1: On-pack marketing claims to test by claim condition and product category.***

|  |  | **Product Category** | | | | |
| --- | --- | --- | --- | --- | --- | --- |
| Stimulus slide: | **Claim type:** | | Fruit/cereal bars | Fruity chews | Yoghurt snacks | Savoury Puffs |
| 1 | **Contains ‘good ingredient’** | | Made with wholegrains | Only sweetened with real fruit | Made with the goodness of milk | Contains real vegetables |
| 2 | **Free from ‘bad’ ingredient** | | Nothing artificial | Preservative free | Free from additives | No nasties |
| 3 | **Child-related claim** | | Right texture to encourage chewing | Encourages self-feeding | Developed with nutritionists | Perfect for little hands |
